# The effect of histopathologic analysis and tissue cultures on inpatient management of cellulitis: a randomized control trial

**DOI:** 10.1007/s00403-024-03224-5

**Published:** 2024-07-23

**Authors:** Michael Lause, Emma Hansen, Karissa Libson, Cory Pettit, Sonia Himed, Kyle P. Rismiller, Sara Huff, Abraham M. Korman, Alecia M. Blaszczak, Willa Hsueh, Nima Milani-Nejad, Leah Kofmehl, Courtney Hebert, Jeffrey M. Caterino, Henry E. Wang, Vedat Yildiz, John C. Trinidad, Catherine G. Chung, Benjamin H. Kaffenberger

**Affiliations:** 1 Department of Dermatology, The Ohio State University Wexner Medical Center, Columbus, OH USA; 2 College of Medicine, The Ohio State University, Columbus, OH USA; 3 Department of Dermatology, University of California Los Angeles, Los Angeles, CA USA; 4 Department of Internal Medicine, The Ohio State University, Columbus, OH USA; 5 Department of Infectious Disease, The Ohio State University, Columbus, OH USA; 6 Department of Emergency Medicine, The Ohio State University, Columbus, OH USA; 7 Department of Biomedical Informatics, The Ohio State University, Columbus, OH USA; 8 Department of Pathology, The Ohio State University, Columbus, OH USA; 9 1328 Dublin Road, Suite 100, Columbus, OH 43215 USA

**Keywords:** Analysis, Biopsy, Cellulitis, Culture, Histopathologic, Inpatient, Management, Pseudocellulitis, Tissue

## Abstract

Background: In the absence of a gold-standard diagnostic modality for cellulitis, sterile inflammatory disorders may be misdiagnosed as cellulitis. Objective: To determine the utility of skin biopsy and tissue culture for the diagnosis and management of patients admitted with a diagnosis of presumed cellulitis. Design: Pilot single-blind parallel group randomized controlled clinical trial in 56 patients with a primary diagnosis of presumed cellulitis. In the intervention group only, skin biopsy and tissue culture results were made available to the primary care team to guide diagnosis and management. Length of hospital stay and antibiotic use were evaluated as outcome measures. Results: Length of stay showed the greatest opportunity for further study as a primary outcome (intervention: 4, IQR (2–6) vs. control: 5 IQR (3–8) days; *p* = 0.124). Limitations: The COVID-19 pandemic placed limitations on participant enrollment and study duration; in addition, data was collected from a single medical center. Conclusion: This study demonstrates that length of stay and anti-pseudomonal antibiotic de-escalation are endpoints that may be influenced by biopsy and tissue culture results in presumed cellulitis patients; these outcomes warrant further study.

## Introduction

Every year, fourteen million cases of cellulitis are treated in the United States, with associated outpatient costs reaching over $3.7 billion [1, 2]. More than 500,000 of these cases lead to inpatient hospitalization; the number of hospitalizations increased by more than 75% from 1998 to 2013 [2]. Readmissions are also common, with an approximate 10% 30-day readmission rate [3]. Cellulitis is typically a clinical diagnosis made in the setting of cutaneous erythema, swelling, pain, and/or warmth. However, no gold-standard diagnostic modality is available. Other cutaneous conditions, categorized generally as pseudocellulitis, can cause inflammation that mimics cellulitis, often prompting unnecessary antibiotic use. For example, atopic dermatitis was found to be a frequent mimicker of cellulitis in a United Kingdom study [4]. Stasis dermatitis and lipodermatosclerosis, which frequently present as antibiotic-refractory bilateral lower extremity erythema, are other common diagnoses that can mimic cellulitis [4].

Inpatient dermatology consultation has been shown to play an impactful role in cellulitis management. A large, multi-institution study demonstrated that 74.3% of cellulitis cases were ultimately diagnosed as pseudocellulitis upon dermatology evaluation [5]. A prospective cohort study found an association between dermatology consultation and decreased length of hospital stay and antibiotic administration in patients with cellulitis and pseudocellulitis [6]. A randomized clinical trial found that dermatology consultation recommendations for cellulitis patients were often related to underlying risk factors, which if treated, could subsequently reduce the risk of recurrent cellulitis [7]. Despite these advantages, many hospitals throughout the United States lack consistent access to inpatient dermatology services. While consultative teledermatology may be a potential tool to close this access gap, there are challenges associated with this modality of care delivery, including disagreements about diagnoses between providers, and a lack of implementation of consult recommendations by primary teams [8–10].

In order to improve hospital care for cellulitis patients, particularly those unable to be evaluated by dermatology consultation, a gold standard diagnostic modality is needed. Prior studies have shown that tissue cultures from skin biopsies are often negative in patients presenting with cellulitis; therefore, the general consensus is that these cultures lack substantial diagnostic or therapeutic benefit [11–13]. Despite the low diagnostic yield of tissue cultures from skin biopsies, we propose that negative cultures can serve an important purpose in patient care, by guiding safe de-escalation of antibiotics and reductions in length of hospitalization, even in the absence of a confirmed alternative, non-infectious etiology. The objective of this study was to determine the utility of skin biopsies and tissue cultures in differentiating cellulitis from pseudocellulitis, and to examine associated outcomes such as length of stay and antibiotic de-escalation.

## Materials and methods

### Design

A single-blind parallel group randomized controlled clinical trial was performed on patients with a primary admission diagnosis of presumed cellulitis or skin and soft tissue infection.

### Population

Eligible patients were adults (18 years or older) admitted to the inpatient internal medicine ward with a primary diagnosis of suspected cellulitis (based on the patient’s overall clinical picture) that did not have features of purulence or necrosis. The following exclusion criteria were applied: any associated surgery to the affected area in the last 90 days, malignancy (including active solid organ and hematologic cancers; non-melanoma skin cancers were permitted), currently pregnant or breast feeding, facial involvement, or any visible skin lesion within 10 cm of the affected area (such as laceration and puncture-related trauma). In addition, incarcerated individuals and direct hospital transfers were excluded.

### Intervention

Eligible patients were screened daily by the research coordinator via medical record number searches. After consent, participants were assigned a unique patient number and randomized to one of two groups using a random number generator: the intervention group or the standard of care (control) group.

For all patients in both groups, encrypted photographs of affected skin areas were taken. Subsequently, three 4–6 mm punch biopsies were obtained from the most proximal portion of erythema. The first biopsy was sent for histopathologic analysis by a dermatopathologist, utilizing standard histopathologic stains including Gram, Grocott’s methenamine silver, and Ziehl-Neelsen (for acid-fast bacteria). Ziehl-Neelsen stains were only performed if the dermatopathologist had suspicions based on the histopathology findings. The second biopsy was sent for tissue culture (bacterial, fungal, and anaerobic). The third biopsy was flash frozen for corollary analysis of gene expression levels in the adipose tissue of cellulitis patients compared to healthy controls. The corollary results are discussed in a separate analysis. Biopsies were performed by the dermatology team, regardless of whether or not dermatology inpatient services were consulted.

The intervention group had skin biopsy and tissue culture results released in the electronic health record (EHR), allowing the primary medical team to monitor pending cultures for positivity. Thus, the intervention itself was based on the availability of culture results to the primary medical team. The standard of care group did not have skin biopsy or tissue culture results reported in the EHR unless medically necessary. For standard of care patients, it was deemed medically necessary to release culture results to the primary team if they returned positive, to allow for the initiation of appropriate and targeted antimicrobial coverage for the identified organism(s).

The patients and dermatopathologist were blinded to group assignments. Hospitalists were aware of the study and agreed to patient participation, but were not explicitly informed of randomization assignments, unless they requested this information. Unblinding occurred based on culture results; hospitalists received mandatory notifications if cultures returned positive, regardless of patient randomization assignment. Hospitalists were able to request a standard inpatient dermatologic consultation at any time if deemed appropriate for patient management. However, dermatology consultations, assessments, and diagnoses were not included as a component of the study protocol. Following discharge, data on outcome measures including length of stay, antibiotic type and duration of use, re-hospitalization rates, and skin biopsy and tissue culture results were collected by the research coordinator. Patients were contacted by phone 30 days after hospital discharge to inquire about additional antibiotic use, hospitalizations, and post-discharge medical care.

### Outcomes

Patient demographics were examined to determine the effect of randomization. Data analysis was performed in an intention-to-treat format, without accounting for laboratory errors, unblinding, and/or patients leaving the hospital against medical advice. The primary outcome measure was length of hospital stay. Antibiotic de-escalation from broad-spectrum coverage to targeted therapy (defined as length of use (days) of intravenous (IV) Methicillin-resistant *Staphylococcus aureus* (MRSA) coverage (vancomycin, daptomycin) and IV *Pseudomonas* coverage (carbapenems, anti-pseudomonal penicillins, ceftazidime and 4th generation cephalosporins, ciprofloxacin and levofloxacin)) was a secondary outcome measure. Additional secondary outcome measures included length of antibiotic use (IV and oral), deaths, 30-day readmission rates, and associations of culture positivity.

### Statistics

Based on National Institutes of Health (NIH) guidelines, as a pilot study, we determined that 60 patients were needed to assess patient flow, effect magnitude, and exploratory hypothesis analysis, while remaining within budget constraints. Ultimately, we enrolled 56 participants prior to pauses on human trial research during the COVID-19 pandemic, and funding lapses.

Descriptive statistics are reported as mean ± standard deviation (std dev), median (interquartile range (IQR)), or total number (%). Normality of data was assessed using visual inspection and the Shapiro Wilk normality test. Continuous data, such as length of stay and duration of antibiotics, were analyzed using a t-test (for normally distributed data) and a Wilcoxon Rank-sums test (for skewed data). Categorical data, such as re-hospitalization within 30 days, were analyzed using a chi-squared or Fisher exact test, as appropriate. The significance level was set to α ≤ 0.05. The data were analyzed using JMP 14PRO and Statistical Analysis Software, version 9.4 (SAS Institute Inc., Cary, NC, USA).

### Study approval

This study was conducted exclusively at The Ohio State University Wexner Medical Center. Institutional Review Board (IRB) approval was obtained (OSU:2018H0147), funding was provided internally through a patient safety advancement grant, and the trial was published on clinicaltrials.gov (NCT03785834). All patients provided written informed consent prior to their participation. Enrollment occurred from August 2018 through March 2020.

## Results

A total of 68,481 patients were electronically screened at admission for admission L03.* ICD-10 codes or a combination of non-specific rash or edema codes (R21.*, R60.*), in combination with antibiotic prescriptions and admission documentation favoring skin and soft tissue infection. Patients were then further screened against study criteria; ultimately, 56 patients met criteria and consented to study participation. Of these 56 patients, 31 and 25 were assigned to the intervention and standard of care groups, respectively. Demographic data for each group is reported in Table 1. There were no significant differences in culture positivity or total duration of antibiotic use between groups. In addition, there were no significant differences in histopathological analysis between groups. The majority of histopathology results were indicative of mild perivascular inflammation, and histopathology failed to identify infection in any patients with negative cultures. Notably, 3 of 56 patients (5%) were diagnosed with bacteremia at the time of admission.

**Table 1:** Patient demographics

Demographic Variable	Value	Control Group	*N* = 25	Intervention Group	*N* = 31	*p*-value
Age (years)	Mean (Std Dev)	55.5 (15.14)	25	48.3 (13.6)	31	0.07
Female	%	44	11	32.3	10	0.41
Race						0.43
Black	%	4	1	6.5	2	
Other	%	0	0	9.7	3	
White	%	96	24	83.9	26	
BMI*	Mean (Std Dev)	37.8 (12.9)	25	36.4 (13.5)	31	0.69
Temperature* (F)	Mean (Std Dev)	98.4 (0.98)	25	98.2 (0.50)	31	0.28
Heart Rate* (bpm)	Mean (Std Dev)	96.0 (14.7)	25	92.1 (16.8)	31	0.37
Respiratory Rate* (rpm)	Mean (Std Dev)	18.1 (2.8)	25	17.4 (2.6)	31	0.30
Systolic Blood Pressure* (mmHg)	Mean (Std Dev)	129 (20.7)	25	138.3 (21.1)	31	0.10
Diastolic Blood Pressure* (mmHg)	Mean (Std Dev)	70 (18.9)	25	79.1 (13.4)	31	**0.050**
Oxygen Saturation* (% RA)	Mean (Std Dev)	0.97 (0.03)	25	0.97 (0.03)	31	0.91
Total WBC*^φ^	Mean (Std Dev)	10.8 (4.2)	25	9.6 (3.9)	31	0.27
Neutrophil count*^#^	Mean (Std Dev)	8.3 (4.1)	25	7.4 (3.8)	31	0.39
Final Diagnosis of Cellulitis or Soft Tissue Infection	%	88	22	93.5	29	0.65
Histopath suspicious cellulitis	%	24	6	16.1	5	0.51
+ Tissue Cultures	%	20.0	5	26.0	8	0.75
Aerobic Bacterial +	%	12.0	3	16.1	5	0.72
Anaerobic Bacterial +	%	8.0	2	3.2	1	0.58
Fungal Culture +	%	0	0	6.5	2	0.50
Left Against Medical Advice	%	4.0	1	6.5	2	1.0

* Indicates on admission

^φ^ White blood cell count reference range: 4.9–11.0 × 10^9^/L

^#^ Neutrophil count reference range: 2.5-7.0 × 10^9^/L

Body mass index (BMI), Beats per minute (bpm), Degrees Fahrenheit (F), Histopathology (Histopath), Millimeters of mercury (mmHg), Positive (+), Respirations per minute (rpm), Saturation percentage on room air (% RA), Standard deviation (Std Dev), White blood cell (WBC)

Major outcome measures including length of hospital stay, antibiotic duration, and 30-day readmission rate did not reach statistical significance (Table 2). However, the shorter length of stay in the intervention group revealed an opportunity for future studies to expand upon (intervention: 4, IQR (2–6) vs. control: 5 IQR (3–8) days; *p* = 0.124). Antibiotic de-escalation did not reach statistical significance (*p* = 0.68 for IV MRSA coverage, *p* = 0.22 for IV *Pseudomonas* coverage). In alignment with existing literature, the rate of culture positivity was low overall (23%, entire study population) and in individual groups (20%, standard of care; 26%, intervention). Dermatology consultations were placed for 6 of 56 patients (11%, 3 standard of care; 3 intervention) at the time of hospital admission.

Secondary analyses evaluated the effect of positive tissue cultures on clinical outcomes (Table 3). 30-day readmission rates were not significantly different between patients with positive and negative tissue cultures, respectively (0 IQR (0–3) vs. 0 IQR (0–1); *p* = 0.16). Additional secondary outcome measures, including length of hospital stay and duration of antibiotics, showed no significant differences between patients with positive and negative tissue cultures. The rate of fungal culture positivity was low (*N* = 2). One patient in question was non-immunosuppressed and had comorbid cirrhosis; she presented with upper extremity cellulitis and was discharged prior to culture growth of yeast. This patient accounted for the single death in the participant population during the study period.

**Table 2:** Antibiotic, length of stay, and readmission outcomes

Variable	Level	Control (*N* = 25)	Intervention (*N* = 31)	Total (*N* = 56)	*p*-value
Antibiotics Rx On Discharge (Y/N)	No	8 (32%)	8 (26%)	16 (29%)	0.610
	Yes	17 (68%)	23 (74%)	40 (71%)	
Length of IV MRSA Coverage (days)	Median [IQR] (min, max)	3 [1, 6] (0, 43)	3 [2, 5] (0, 42)	3 [2, 5.5] (0, 43)	0.678
Length of IV *Pseudomonas* Coverage (days)	Median [IQR] (min, max)	2 [0, 5] (0, 43)	0 [0, 3] (0, 19)	1 [0, 4.5] (0, 43)	0.219
Discharge Length of IV Antibiotics (days)*	Median [IQR] (min, max)	0 [0, 0] (0, 32)	0 [0, 0] (0, 14)	0 [0, 0] (0, 32)	0.115
Total Antibiotic Length (days)	Median [IQR] (min, max)	11 [7, 16] (1, 43)	11 [10, 17] (0, 42)	11 [8, 16] (0, 43)	0.590
Length of Stay (days)	Median [IQR] (min, max)	5 [3, 8] (2, 50)	4 [2, 6] (1, 16)	4 [3, 7] (1, 50)	0.124
Readmitted within 30 days from discharge (Y/N)	No	18 (72%)	26 (84%)	44 (79%)	0.281
	Yes	7 (28%)	5 (16%)	12 (21%)	
Number readmitted within 30 days from discharge (n)	Median [IQR] (min, max)	0 [0, 1] (0, 2)	0 [0, 0] (0, 3)	0 [0, 0] (0, 3)	0.394

* Indicates the number of days that a patient continued to receive intravenous antibiotics after discharge

Interquartile range (IQR), Intravenous (IV), Maximum (max), Methicillin-resistant *Staphylococcus aureus* (MRSA), Minimum (min), Prescription (Rx)

**Table 3:** Outcomes based on culture results

Variable	Level	Culture - (*N* = 43)	Culture + (*N* = 13)	Total (*N* = 56)	*p*-value
Length of IV MRSA Coverage (days)	Median [IQR] (min, max)	3 [2, 5] (0, 43)	4 [2, 6] (1, 16)	3 [2, 5.5] (0, 43)	0.531
Length of IV *Pseudomonas* Coverage (days)	Median [IQR] (min, max)	2 [0, 5] (0, 43)	0 [0, 1] (0, 15)	1 [0, 4.5] (0, 43)	0.395
Discharge Length of IV Antibiotics (days)*	Median [IQR] (min, max)	0 [0, 0] (0, 32)	0 [0, 0] (0, 9)	0 [0, 0] (0, 32)	0.842
Total Antibiotic Length (days)	Median [IQR] (min, max)	11 [7, 17] (0, 43)	10 [9, 15] (2, 24)	11 [8, 16] (0, 43)	0.552
Length of Stay (days)	Median [IQR] (min, max)	4 [3, 7] (1, 50)	6 [2, 9] (1, 17)	4 [3,7] (1, 50)	0.814
Readmitted within 30 days from discharge (Yes/No)	No	32 (74%)	12 (92%)	44 (79%)	0.257
	Yes	11 (26%)	1 (8%)	12 (21%)	
Number readmitted within 30 days from discharge (n)	Median [IQR] (min, max)	0 [0, 1] (0, 3)	0 [0, 0] (0, 1)	0 [0, 0] (0, 3)	0.158

* Indicates the number of days that a patient continued to receive intravenous antibiotics after discharge

Interquartile range (IQR), Intravenous (IV), Maximum (max), Methicillin-resistant *Staphylococcus aureus* (MRSA), Minimum (min), Negative (-), Positive (+)

**Table 4:** Culture data

Organism	Control^a^	Intervention^a^	Total^a^
**Anaerobic Bacteria**	2	1	3
*Cutibacterium acnes*	1	0	1
*Peptinophilus harei*^*b*^	1	0	1
*Campylobacter ureolyticus*^*b*^	1	0	1
*Finegoldia* species	0	1	1
**Aerobic Bacteria**	3	5	8
*Streptococcus agalactiae*^*c*^	0	2	2
*Escherichia coli*^*d*^	1	0	2
*Alcaligenes faecalis*^*d*^	1	0	1
Methicillin-sensitive *Staphylococcus aureus*^*c*^	0	2	2
Methicillin-resistant *Staphylococcus aureus*	1	2	2
*Enterococcus faecalis*	1	0	1
**Fungal**	0	2	2
*Pithomyces* species	0	1	1
*Cryptococcus neoformans*	0	1	1

^a^ Data corresponds to the number of patients

^b^ Same patient was culture-positive for both *P. harei* and *C. ureolyticus*

^c^ Same patient was culture-positive for both *S. agalactiae* and Methicillin-sensitive *S. aureus*

^d^ Same patient was culture-positive for both *E. coli* and *A. faecalis*

## Discussion

We examined the role of skin biopsies and tissue cultures in patients admitted with presumed cellulitis. Our data provides unique insights regarding the evaluation and management of patients with cellulitis and pseudocellulitis. Although we were unable to complete the planned enrollment and our clinical outcome measures did not reach statistical significance, this study serves as an effective pilot supporting the feasibility of future studies. The observed trends in length of hospital stay and IV anti-pseudomonal antibiotic de-escalation suggest that expansion of our methods to a larger, multicenter approach is warranted.

Here, we corroborate the low rate of positive tissue cultures in cellulitis [11–13]. Interestingly, low rates of organism detection have been demonstrated in cellulitis patients when molecular methods are used as well, pointing to a potential pathophysiological underpinning for these findings [14]. However, we emphasize the utility of negative cultures in cellulitis management, as primary hospital teams may be more likely to discharge patients or deescalate antibiotics based on negative cultures. It is important to recognize that all patients included in this study received antibiotics in the emergency department prior to admission, which may also have affected the culture results. For rare cellulitis patients whose tissue cultures do yield positive results, this information may help dictate management. For example, positive fungal cultures for one of the patients in this study allowed us to promptly begin appropriate antifungal treatment. Notably, histopathologic analysis for this patient was uninformative, therefore, management was based primarily on culture findings. Finally, similar to the tissue culture results, the rate of bacteremia in this study at the time of admission was low (5%), suggesting that blood cultures also lack diagnostic utility in presumed cellulitis patients.

Secondary analyses showed that patients with positive tissue cultures trended toward a lower 30-day readmission rate compared to those with negative tissue cultures. It is plausible to suspect that culture-positive patients with identified organism(s) and available antibiotic susceptibilities were able to be treated with a more targeted antibiotic regimen, which may have reduced the risk for readmission. Alternatively, it is possible that culture-negative patients were actually patients with pseudocellulitis (sterile inflammatory conditions mimicking cellulitis) who experienced flares or reoccurrences requiring subsequent hospitalizations.

This study has limitations. First, patients did not routinely receive an expert diagnostic assessment by dermatology to confirm the diagnosis of cellulitis or the likelihood of pseudocellulitis, and elective dermatology consultations were placed for only 6 of 56 patients. This is an important issue to address, as cellulitis may be overdiagnosed in the absence of dermatology inpatient consultation [5, 15]. Prior studies have demonstrated associations between dermatology involvement and diagnostic corrections from cellulitis to pseudocellulitis; in addition, hospitals without dermatology access have been shown to code more cutaneous diseases as cellulitis compared to those with dermatology access [5, 15]. Due to their low diagnostic yield, biopsies and tissue cultures are not an adequate substitution for inpatient dermatologist consultation, however, poor availability of dermatology consultation services remains a substantial barrier. These issues highlight a substantial need for a scalable gold-standard testing methodology for the clinical treatment and study of cellulitis.

The sample size and duration of this study were limited due to restrictions imposed by the COVID-19 pandemic, thus, enrollment was unable to be completed. In addition, data collection occurred at a single academic medical center, placing limitations on the recruitment population. Based on inclusion and exclusion criteria, we did not assess post-surgical or cancer patients, facial cellulitis, puncture or laceration-related trauma, or prisoners, further limiting the generalizability of our results.

Regarding methodology, several instances of unblinding occurred throughout the study – these were both necessary (due to positive tissue culture results in standard of care patients that required medical management) and unnecessary (related to laboratory reporting errors). Positive tissue culture results in the standard of care group that required unblinding may have skewed our results. In addition, 6.5% and 4.0% of patients in the intervention and control groups, respectively, left the hospital against medical advice and were unable to be assessed further. Prior studies have demonstrated similar difficulties in care continuity for cellulitis patients [16]. Future studies should account for these methodological challenges.

Finally, the interpretation of our results is limited by confounding infectious sequelae that were discovered in some patients during hospitalization, including osteomyelitis (5 patients, diagnosed clinically without bone biopsy) and endocarditis (1 patient). As a result, these patients required alterations in their antibiotic regimens that were unrelated to cellulitis status. In addition, one of the patients with osteomyelitis required a significantly extended course of broad-spectrum IV antibiotics (43 days of MRSA and *Pseudomonas* coverage), which was an outlier finding compared to the rest of the cohort. Future studies should control for the development of infectious sequelae beyond cellulitis to avoid potential confounding.

## Conclusions

This pilot study evaluated the feasibility and utility of skin biopsy and tissue culture in patients admitted with a diagnosis of presumed cellulitis. Our preliminary findings demonstrate that length of hospital stay and anti-pseudomonal antibiotic de-escalation are endpoints that warrant further exploration. Larger, multicenter studies are needed to better characterize the utility of skin biopsy and tissue cultures in cellulitis diagnosis and management, as well as the associated clinical and economic implications.

## Data Availability

The authors confirm that the data supporting the findings of this study are available within the article.

## Declarations

### Prior publications

Early iterations of this work were previously published in pre-print format on Research Square (2022, doi:10.21203/rs.3.rs-1674539/v1) and submitted to Archives of Dermatological Research (returned with reviewer comments provided). The current manuscript represents an update of prior iterations with revisions and additional data.
